# Gut Microbiota and Food Allergy: A Review of Mechanisms and Microbiota-Targeted Interventions

**DOI:** 10.3390/nu17183009

**Published:** 2025-09-20

**Authors:** Roxana Cristina Mareș, Maria Oana Săsăran, Cristina Oana Mărginean

**Affiliations:** 1 Department of Pediatrics 1, George Emil Palade University of Medicine, Pharmacy, Sciences and Technology of Târgu Mureș, Gheorghe Marinescu 38, 540136 Târgu Mureș, Romania; cristina_campean2005@yahoo.com (R.C.M.); marginean.oana@gmail.com (C.O.M.); 2 Department of Pediatrics 3, George Emil Palade University of Medicine, Pharmacy, Sciences and Technology of Târgu Mureș, Gheorghe Marinescu 38, 540136 Târgu Mureș, Romania

**Keywords:** gut microbiota, food allergy, mechanisms, microbiota-targeted interventions, children

## Abstract

**Background**: Food allergies (FAs) have become a major public health concern worldwide, with rising prevalence particularly among children. Traditional genetic and allergen exposure models do not fully explain this increase, prompting growing interest in the role of the gut microbiota. Early-life microbial colonization is now recognized as a critical determinant of immune development, with disruptions in microbial balance implicated in allergic sensitization. **Objective**: This review aims to synthesize recent human studies investigating the relationship between gut microbiota composition and food allergies, with an emphasis on underlying mechanisms and the potential of microbiota-targeted interventions. **Methods**: A literature search was conducted, including eligible studies concerning gut microbiota and food allergy. A total of 31 studies met the inclusion criteria. **Results**: The findings indicate that early-life factors, including delivery mode, feeding practices, antibiotic exposure, and environmental microbial diversity, have a significant influence on gut microbial colonization. Allergic children consistently exhibit reduced microbial diversity and lower levels of beneficial taxa such as *Bifidobacteria*, *Faecalibacteria*, and *Clostridia*. Microbial dysbiosis is associated with enhanced type 2 immune responses, reduced regulatory T cell activity, and altered profiles of short-chain fatty acids. Dietary modulation through prebiotics, probiotics, and synbiotics has shown potential in restoring microbial balance and promoting immune tolerance, although with varying degrees of efficacy depending on the strains, formulas, and timing of intervention. **Conclusions**: The gut microbiota plays a central role in the pathogenesis and potential prevention of food allergies. Microbiota-targeted dietary strategies, particularly in early life, offer promising avenues for promoting immune tolerance.

## 1. Introduction

Food allergy (FA) is a recurrent atopic disorder that occurs after exposure to specific food allergens. The allergy can manifest through mild symptoms such as diarrhea or vomiting, but it can also lead to severe adverse reactions, including anaphylactic shock and even death [1]. This condition represents a growing public health problem, affecting approximately 8–10% of children and 4% of adults globally [2,3], with a higher prevalence in urban areas compared to rural ones [4,5,6]. In recent decades, the incidence of FA has increased significantly, a phenomenon also reflected in the rise in hospitalizations for food-induced anaphylaxis, which has been described as “the second wave of the allergy epidemic” following the increase in asthma and respiratory allergies [7,8].

This rapid and global increase in prevalence suggests a significant involvement of environmental factors in the development of food allergies, especially in the context of modern lifestyle changes. Factors such as birth methods [9,10], breastfeeding [11,12,13,14], dietary patterns [15], antibiotic use [16], vaccinations [17], and exposure to pathogens [18] have the potential to influence the composition and diversity of the gut microbiota. Increasing experimental and epidemiological evidence led to the hypothesis that intestinal bacteria may play a central role in the development of food allergies.

Early microbial colonization of the intestine, particularly in the first 6 months of life, is essential for the development of a tolerant immune system. Microbial imbalances—such as reduced diversity or an increased *Enterobacteriaceae*/*Bacteroidaceae* ratio—have been associated with increased susceptibility to food sensitization [19]. Furthermore, children born by cesarean section (C-section), who are not exposed to maternal vaginal microbiota, have a higher risk of developing allergic conditions [10].

The gut microbiota has an essential role in maintaining the integrity of the intestinal barrier [20], as well as in modulating the inflammatory microenvironment within the intestine. Moreover, the modulation of the intestinal microbiota—from prebiotics and probiotics to fecal microbiota transplantation (FMT)—opens up new possibilities for treating conditions with an increasing prevalence and social impact [21,22,23].

In this context, the present article aims to review the most recent articles on this topic and analyze the interaction between gut microbiota and food allergies, with an emphasis on the mechanisms involved and potential implications for prevention and treatment.

## 2. Materials and Methods

C.R.M. and M.O.S., under the supervision of C.O.M., performed a comprehensive literature search across three major electronic databases—PubMed, Web of Science, and Scopus—to identify relevant studies published from January 2015 onwards. The focus was on the relationship between gut microbiota and food allergies in pediatric populations. The search strategy employed a combination of keywords such as “food allergies”, “gut microbiota”, “cow’s milk allergy”, “peanut allergy”, “intestinal permeability”, “leaky gut”, “gut dysbiosis”, “gut microbiome”, “microbial diversity”, “early-life colonization”, and “immune tolerance”. Boolean operators (AND, OR) were used to refine and optimize the search sensitivity.

Eligible studies included only full-text articles published in English that followed randomized controlled trials (RCTs), cohort, cross-sectional, or longitudinal designs. To ensure a focus on primary empirical evidence, we excluded reviews, meta-analyses, case reports, editorials, conference abstracts, non-English publications, and duplicate records.

### Quality Assessment

In this review, we evaluated the methodological quality of all 31 included studies using the NIH Study Quality Assessment Tools [24]. Each tool was applied in its entirety based on the specific study design (e.g., observational, cross-sectional, or randomized controlled trial), with individual items rated as “Yes”, “No”, “Not Reported”, “Not Applicable”, or “Cannot Determine”. Studies receiving 11 or more “Yes” responses and showing no significant methodological flaws were categorized as good quality. Those with 7 to 10 “Yes” responses, along with 3 to 6 “No” or “Not reported” responses—typically due to limitations such as small sample size or other methodological issues—were deemed to be of fair quality. None of the included studies were classified as poor quality. This quality grading system facilitated a structured interpretation of the strength and reliability of the findings across various study designs (see Table 1).

## 3. Results

**Figure 1:**
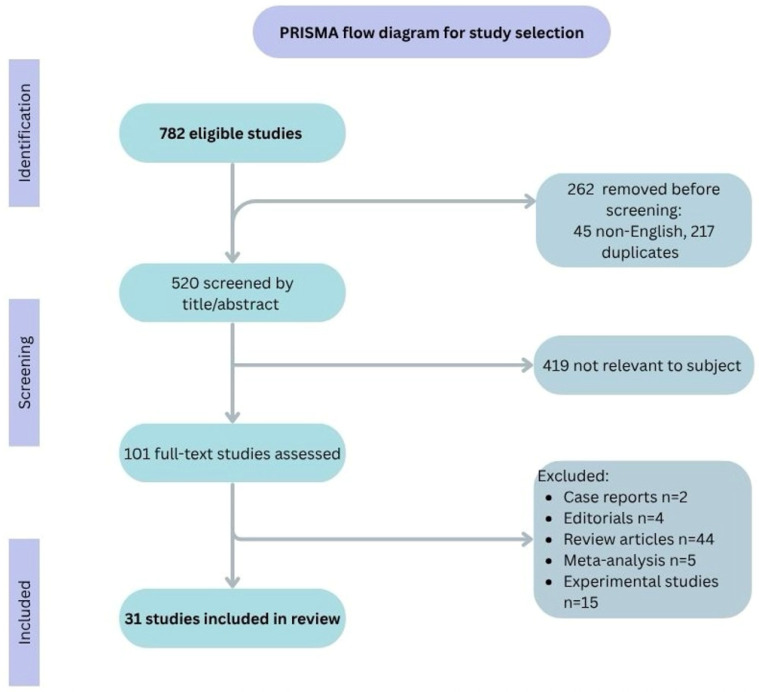
PRISMA flow diagram for assessment of eligible studies.

The initial search retrieved 782 records, which we narrowed down to 520 after eliminating duplicates and non-English entries (Figure 1). The entire selection process is illustrated in Figure 1, following the PRISMA 2020 guidelines and revised flow diagrams [54]. Of these, 419 articles were excluded because they lacked relevance to the role of gut microbiota in allergy development or its modulation through therapeutic interventions. After further excluding experimental studies, meta-analyses, reviews, case reports, and editorials, 31 studies met the inclusion criteria. These selected publications specifically addressed the involvement of the gut microbiota in the pathogenesis of food allergies and investigated dietary, prebiotic, probiotic, or synbiotic approaches aimed at modulating the microbiota to influence allergic outcomes.

Table 2 summarizes the observational studies included in the review, which highlight the associations between gut microbiota and food allergy development. Table 3 presents the experimental studies included, which investigated microbiota-targeted interventions in this context.

The collective evidence from the observational studies included demonstrates that the development, persistence, or resolution of food allergy in childhood is closely linked to the composition and functional capacity of the gut microbiome. A recurring finding is that children with food allergies tend to exhibit reduced microbial diversity and richness compared to healthy controls, indicating that a less complex microbial ecosystem is associated with an increased risk of sensitization and allergic disease [32,39,43,47,50]. Specific bacterial shifts have also emerged consistently: taxa generally considered protective, such as Bifidobacterium, Lactobacillus, Clostridia clusters IV and XIVa, and Faecalibacterium prausnitzii, are often diminished in allergic individuals [11,12,35,36,39,40,42,45,52], whereas potentially pathogenic or pro-inflammatory groups, including Escherichia-Shigella, Ruminococcus gnavus, Enterococcus, Eggerthellaceae, and members of the Erysipelotrichaceae family, are enriched [30,36,39,43,44,46,47,50]. These alterations in microbial profiles translate into functional consequences, such as reduced production of short-chain fatty acids (SCFAs), impaired fiber degradation, and enhanced pro-inflammatory signaling, which collectively favor T2 immune polarization and diminished regulatory T cell (Treg) responses [32,36,50].

Importantly, several studies highlight that these microbial signatures can be detected early in infancy, well before the clinical manifestation of food allergy, suggesting a predictive role of the microbiota in shaping immune trajectories [12,26,42,52]. Factors such as mode of delivery, feeding practices (particularly breastfeeding versus formula feeding), timing of allergenic food introduction, antibiotic exposure, dietary diversity, and family environment (e.g., the presence of siblings) all significantly influence the establishment and maturation of the gut microbiome, thereby modulating allergy risk [11,12,35,40,45,52]. For instance, breastfeeding is strongly associated with enrichment of Bifidobacterium and Lactobacillus [11,12,35,45], while cesarean delivery and antibiotic exposure are linked to delayed microbial maturation and increased susceptibility to allergic outcomes [12,52]. Children with microbiota profiles which are enriched in tolerance-promoting taxa, such as Bifidobacterium and Clostridia, are more likely to develop immune tolerance and even outgrow early-life food allergies [12,42,44], whereas dysbiotic patterns characterized by dominance of Escherichia-Shigella or depletion of SCFA producers predict persistent food allergy [39,44].

The experimental studies included in Table 3 collectively highlight that targeted dietary interventions, particularly the use of specialized formulas supplemented with probiotics, prebiotics, or synbiotics, as well as oral immunotherapy (OIT) combined with microbiota-modulating strategies, can significantly influence allergy outcomes in infants and children with cow’s milk allergy (CMA) and other IgE-mediated conditions.

A strong body of evidence supports the benefits of extensively hydrolyzed casein formula (EHCF) supplemented with Lactobacillus rhamnosus GG (LGG). Several large clinical trials [27,29,34,53] consistently showed that EHCF + LGG reduced the incidence of other allergic manifestations (eczema, asthma, urticaria, rhinoconjunctivitis), accelerated the acquisition of immune tolerance to cow’s milk proteins, and decreased the risk of functional gastrointestinal disorders later in childhood.

Other formula-based interventions demonstrated microbiome-modulating benefits. A synbiotic-containing amino acid formula [28] increased fecal Bifidobacteria levels to levels comparable with breastfed infants, while supplementation with Bifidobacterium bifidum TMC3115 [31] reduced allergic symptoms across multiple systems, lowered pro-inflammatory cytokines, increased IL-10, and improved gut microbial diversity. Similarly, formulas supplemented with human milk oligosaccharides (HMOs, e.g., 2′FL, LNnT) shifted the gut microbiome toward breastfed infant-like profiles, increased Bifidobacterium, decreased potential pathogens, and enhanced SCFA production [37].

Several studies evaluated oral immunotherapy (OIT) combined with probiotics. Hanada et al. [38] found that adding Lactiplantibacillus plantarum to OIT increased the rate of milk tolerance and improved immune biomarkers (e.g., higher IgG4, lower IL-5/IL-9). Shibata et al. [49] showed that higher baseline Bifidobacterium dominance in gut microbiota predicted greater success in achieving sustained unresponsiveness during milk OIT, underscoring the microbiota’s role in therapy outcomes.

Preventive strategies targeting mothers and neonates also showed promise. Probiotic supplementation with Escherichia coli EcO83 in newborns of allergic mothers [41] reduced allergy incidence at 10 years, likely through immune modulation (increased IL-10, CD83 expression). Maternal prebiotic supplementation during pregnancy [48] altered both maternal and infant microbiota, enriched Bifidobacterium, reduced Negativicutes, and increased maternal acetic acid levels, indicating beneficial metabolomic shifts. By contrast, Dawson et al. [33] showed that a dietary counseling intervention in pregnant women improved maternal dietary variety but did not significantly alter maternal or infant microbiota, suggesting that supplementation may have stronger effects than diet alone.

In summary, these experimental studies demonstrate that microbiota-targeted interventions—particularly probiotic-supplemented formulas (EHCF + LGG, HMO-enriched formulas), probiotic therapy combined with OIT, and maternal/neonatal supplementation—can positively influence immune tolerance acquisition, reduce allergic manifestations, and shape a gut microbiota profile more similar to that of breastfed or non-allergic infants. The evidence consistently points to Bifidobacterium enrichment, increased microbial diversity, and higher SCFA production as common protective signatures linked with improved clinical outcomes and accelerated tolerance development in CMA and related allergic conditions.

**Figure 2:**
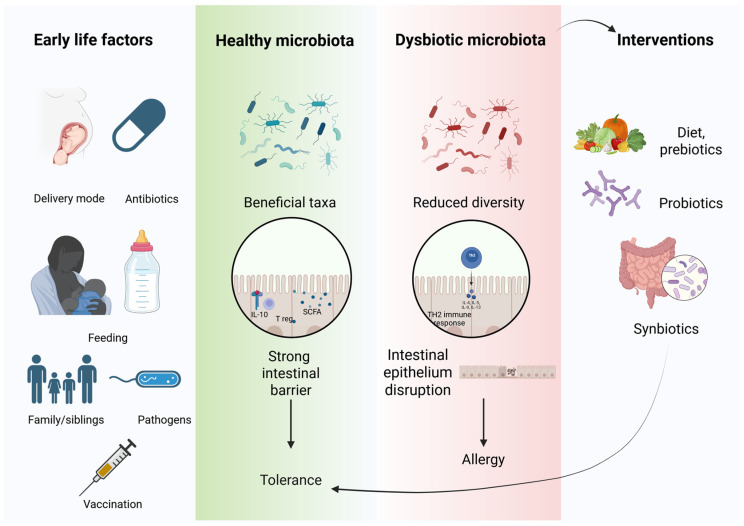
Summary of early life factors and interventions which can modulate the microbiome, a key player in the development of allergies. Created in BioRender. Sasaran, M. (2025) https://BioRender.com/tg5kb0s, accessed on 12 September 2020. Legend: IL—interleukin; Th—T helper cell; T reg—regulatory T cell; SCFA—short-chain fatty acids.

A summary of the interplay between early life factors and microbiota as determinants/modulators of the intestinal barrier integrity and subsequent immune response activation has been visually represented through Figure 2. Moreover, the figure also depicts potential interventions (diet, probiotics, prebiotics and synbiotics) that can regulate the microbiota composition and have a beneficial effect in subjects with food allergy.

## 4. Discussion

### 4.1. Hygiene Hypothesis

The hygiene hypothesis, initially proposed by David Strachan in 1989, posits that exposure to mild infections in early life can have a protective effect against allergic diseases [55]. This idea was inspired by epidemiological observations that showed a lower incidence of allergic rhinitis and eczema among children from larger families, suggesting that interaction with older siblings contributes to a more balanced maturation of the immune system [56].

The microbiota hypothesis further developed this idea, focusing on the changes in microbiota secondary to the influence of the modern Western environment, characterized by increased hygiene, processed foods, and reduced contact with environmental microorganisms. An early alteration of the gut microbiota can compromise the development of immunological tolerance and increase the likelihood of allergic disease onset [57].

Observational data support this hypothesis. For example, pre- and postnatal exposure to pets, vaginal birth, and childhood spent in rural environments reduce the risk of developing allergic diseases [58,59].

Regarding FA, a study including 5276 children showed that the presence of older siblings and dog ownership were associated with a significant decrease in the risk of egg allergy [60]. Another study involving infants from Australian and Danish cohorts [39] found that having siblings in early life significantly reduces the risk of developing FA, a protective effect mediated by accelerated gut microbiota maturity, characterized by higher microbial alpha diversity and increased short-chain fatty acids (SCFAs) such as acetate and butyrate. Infants with siblings exhibited a more mature gut microbiome and higher levels of key SCFAs at 1, 6, and 12 months of age. A higher gut microbiota maturity index (MAZ score) at 6 and 12 months was independently associated with a lower risk of food allergy and polysensitization.

The initial gut microbial configuration and its ability to recover after disturbances are critical determinants of allergy risk [52]. Infants born during the pandemic-related social distancing period showed higher levels of *Bifidobacteria* and lower levels of environmental bacteria, such as *Clostridia* [45]. Additionally, higher *Bifidobacteria* levels at 6 months and a greater relative abundance of butyrate producers at 12 months were associated with a lower incidence of atopic dermatitis. The low use of antibiotics and high prevalence of breastfeeding in the study cohort likely contributed to higher levels of *Bifidobacteria*. Moreover, the presence of siblings was associated with significantly higher levels of *Bifidobacteria* and a reduced risk of allergic diseases, suggesting a protective effect.

Over the past 30 years the Hygiene hypothesis has evolved significantly. The discovery of Tregs, innate lymphoid cells (ILCs) and their diverse roles in immune regulation challenge the older T1/T2 dichotomy and illustrate more nuanced mechanisms of immune education by microbes. Also, epigenetic mechanisms (DNA methylation, histone modification, microRNAs) are increasingly recognized as key mediators translating environmental exposures—such as farm dust or raw milk consumption- into lasting immunological effects, supporting or protecting against allergic disease [61,62].

### 4.2. Development of Gut Microbiota in Children and Adults

The human gut microbiota represents a highly diverse community of microorganisms, including bacteria, archaea, viruses, and eukaryotes, that coexist synbiotically in the gastrointestinal tract [63]. Among these, bacteria from the *Firmicutes* and *Bacteroidetes* phyla dominate the intestinal ecosystem, accounting for over 90% of the total intestinal flora [64]. Most of these bacteria are strict anaerobes, outnumbering aerobes by approximately 1000 times [65].

The process of microbial colonization begins immediately after birth, influenced by the mode of delivery (vaginal or C-section), the type of feeding, and the surrounding environment [9,12]. Newborns are initially colonized by microorganisms from their mother—from the skin, vagina, feces, and especially from breast milk, which is a significant source of beneficial bacteria [66].

In the first three months of life, *Bifidobacteria* and *Lactobacilli* are the dominant genera, with an essential role in immune development and in preventing colonization by pathogenic bacteria [67].

In contrast, children born by C-section exhibit a reduced number of *Bacteroidetes* and *Escherichia coli* bacteria, as well as reduced microbiota biodiversity, changes that persist for at least up to two years of age [68]. Additionally, colonization with pathogenic bacteria, such as *Klebsiella*, *Enterococcus*, and *Clostridium*, is more frequent in children born via C-section [69,70]. Epidemiological studies have confirmed a higher incidence of food allergies and bronchial asthma in these children [71].

Breastfeeding is the most significant dietary factor influencing the infant’s intestinal microbiome, leading to higher levels of beneficial bacteria, such as *Bifidobacteria*, *Lactobacilli*, and *Clostridia* [11]. However, exclusively breastfed infants born via C-section also exhibited reduced gut microbiota diversity and reduced levels of *Bacteroides* at 3 months [12].

Antibiotic treatment is another factor that modifies the gut microbiota. Studies on children treated with antibiotics have shown that their microbiota is less diverse, with a lower number of *Actinobacteria*, *Lactobacilli*, and *Bacteroides* [72]. A meta-analysis of 47 studies demonstrated that exposure to antibiotics in the first 2 years of life is associated with a subsequent increased risk of eczema, allergic rhinitis, and food allergies [73]. Antimicrobial exposure at delivery also significantly altered the gut microbiota composition and overall diversity in early infancy [52].

After weaning, the diversity of the gut microbiota significantly increases as the diet becomes more varied. These changes in nutrition lead to the establishment of new bacterial populations that will characterize the adult microbiota [35]. In adulthood, the most common intestinal bacteria belong to the genera *Firmicutes* and *Bacteroides*, reflecting a balance between the metabolic and immunological functions of the microbiota [74]. In a study by Marrs on breastfed infants, the early introduction of allergenic solids from 3 months significantly increased Shannon microbial diversity at 6 months [12]. However, no evidence was found that specific microbiota changes resulting from the early introduction of allergenic foods were associated with the development of food allergies.

As an individual ages, the composition of the microbiota changes again, with a decrease in the abundance of *Firmicutes* and *Bifidobacteria*, and an increase in the genera *Bacteroides* and *Proteus* [75]. These changes can have a negative impact on intestinal function and overall health. The gastrointestinal tract, however, remains a favorable environment for the development of bacteria, especially facultative and strict anaerobes, which utilize available nutrients to support fermentation, metabolism, and replication processes [76].

### 4.3. Pathophysiological Mechanisms Linking Gut Microbiota to Food Allergy Development

The intestinal mucosal barrier, comprising epithelial lining, mucus layer, microbiota, and associated immune elements, plays a key role in maintaining gastrointestinal homeostasis [77].

Inflammation of the intestinal mucosa triggers a cascade of pathological mechanisms that disrupt the integrity of the epithelial barrier [78]. These mechanisms include the downregulation of proteins associated with tight junctions, as well as dysfunctions in vesicular transport and alterations in the assembly and contractility of the actomyosin cytoskeleton [79]. The cumulative effect of these alterations is the disruption of tight junction integrity and an increase in paracellular permeability—a phenomenon commonly referred to as “leaky gut syndrome” [80]. This enhanced permeability leads to uncontrolled translocation of dietary proteins, toxins, and microbes across the intestinal epithelium, where they can trigger aberrant mucosal immune responses [81].

In the context of food allergies, the passage of allergens through a compromised epithelial barrier triggers the activation of the immune system, leading to the production of allergen-specific IgE and the activation of mast cells and basophils. Upon re-exposure to the allergen, these effector cells release histamine and other pro-inflammatory mediators, further increasing intestinal permeability and perpetuating the inflammatory response [80,82,83]. Conversely, under physiological conditions, intact epithelial tight junctions, a well-structured mucus layer, and balanced microbial colonization allow for controlled antigen sampling by epithelial and immune cells. This regulated antigen uptake favors the induction of oral tolerance, largely through the differentiation of IL-10– and TGF-β-producing regulatory T cells (Treg), which suppress aberrant immune activation against food proteins [77,84]. Thus, the intestinal barrier acts as a key determinant of whether exposure to dietary antigens results in tolerance or sensitization.

The intestinal microbiome supports immune homeostasis through multiple mechanisms, including the production of metabolites with anti-inflammatory effects, the modulation of inflammation-related cells, and the strengthening of the intestinal epithelial barrier. As such, the intestinal microbiome plays a crucial role in preventing FAs, in part by stimulating the production of anti-inflammatory cytokines, such as interleukin-10 (IL-10) [84]. For example, Bacteroides fragilis produces a capsular polysaccharide (PSA) that stimulates the differentiation of CD4+ lymphocytes into IL-10-secreting regulatory T cells (Treg). PSA is effective in preventing and even treating experimental colitis in mice [85].

Similarly, certain Clostridium strains from clusters IV, XIV, and XVIII induce IL-10-producing Treg cells in the colon. The administration of a combination of such strains to murine models of allergic colitis has resulted in symptom amelioration [86]. In human studies, Clostridia are more abundant in the stool samples of children who have outgrown cow’s milk allergy by the age of 8, and are less present between 3 and 6 months in children who later develop food sensitization [87]. Bifidobacteria and Lactobacilli also support the activation of regulatory T cells, which reduce allergic responses and promote the production of anti-inflammatory cytokines, including IL-10 [88].

Microbiota-derived metabolites, particularly SCFAs—butyrate, propionate, and acetate act on free fatty acid receptors—such as FFAR2 (GPR43), FFAR3 (GPR41), and HCAR2 (GPR109A)—expressed on gut epithelial cells and immune cells. These interactions can trigger immune signaling via MAPK pathways and promote oral tolerance mechanisms. SCFAs can induce IL-10 secretion by T1 cells via activation of the GPR43 receptor [89]. Also, SCFAs stimulate the expression of the transcription factor Blimp-1 through the STAT3 and mTOR pathways, thereby increasing IL-10 production. Additionally, butyrate facilitates the differentiation of naive T lymphocytes into IL-10-producing Treg cells. A key molecular action of SCFAs is the inhibition of histone deacetylases (HDACs), leading to enhanced histone acetylation and elevated expression of immune-regulatory genes. Butyrate, in particular, potently inhibits both class I and most class II HDACs. By enhancing histone acetylation at the FOXP3 locus (a master regulator of Treg development), SCFAs facilitate higher FOXP3 expression, which drives Treg differentiation and stability. SCFAs stimulate Treg expansion not only in the colon but also in peripheral tissues like the skin, amplifying systemic immune regulation. Beyond Treg induction, SCFAs dampen type 2 airway inflammation, modulate mast cell development and function, and reduce eosinophil trafficking and survival—all processes central to allergic responses. Thus, SCFAs, through epigenetic and receptor-mediated pathways, play a central role in developing immune tolerance via Treg enhancement and direct inhibition of allergic effectors [90].

Importantly, these mechanisms are most influential during early life, when immune and epithelial systems are still developing. The timing of allergenic food introduction interacts with barrier function and microbiota composition to shape oral tolerance [12]. Early, age-appropriate exposure to allergenic foods during the critical “window of opportunity” in infancy appears to promote tolerance, provided that barrier integrity and microbial colonization are sufficiently mature [91]. Controlled antigen presentation during this developmental window supports Treg induction rather than T2 skewing, thereby reducing the risk of food allergy. By contrast, microbial dysbiosis or barrier dysfunction in infancy may predispose to sensitization upon allergen exposure [92].

Taken together, these findings underscore that the intestinal barrier does not act in isolation but rather interfaces with diet and microbiota to shape immune education. Preserving epithelial integrity, promoting colonization with immunoregulatory microbes, and introducing allergenic foods during a critical immunological window appear to be convergent strategies that support the development of tolerance and reduce the burden of food allergy.

### 4.4. Experimental Evidence from Animal Models

Animal studies have shown that germ-free (GF) mice exhibit a significant reduction in the number of mucus-secreting goblet cells in the cecum, resulting in a less stable mucus layer compared to conventionally colonized mice [93]. In addition, germ-free mice display various structural abnormalities of the intestine, including reduced intestinal surface area, shortened ileal villi, and a decreased number of intestinal crypts—alterations attributed to the absence of microbial influences on intestinal development and architectural maintenance [94].

Further evidence indicates that germ-free mice fail to develop oral tolerance to dietary antigens. This phenomenon can only be reversed if the gut microbiota is reconstituted during the neonatal period [95]. More recently, fecal microbiota transplantation from food-allergic patients into germ-free mice has been shown to confer susceptibility to food allergy [96]. Conversely, colonization with microbiota from healthy infants protected mice from anaphylactic reactions to dietary allergens such as cow’s milk protein [97].

In murine models, specific microbial taxa have been shown to have protective effects against food allergies. For instance, Anaerostipes caccae has been shown to prevent allergic responses to food antigens [98], while Bifidobacterium infantis and *B. lactis* species, commonly found in breastfed infants, have been reported to reduce allergen-specific IgE activity in mice sensitized to shellfish allergens [99].

### 4.5. The Gut Microbiota–Food Allergy Axis: Evidence from Human Studies

Recent studies have identified distinct microbial signatures that differentiate food-allergic individuals from healthy controls. These include a consistent reduction in fecal microbiota richness in allergic individuals, along with lower diacylglycerol levels and higher levels of Phascolarctobacterium faecium and Ruminococcus bromii in healthy twins [32]. Alterations in both the microbiome and metabolome have been correlated with the clinical severity of food allergy [32]. Further microbial imbalances have been observed in children with food allergies, including a decrease in Desulfovibrionaceae and an increase in Bifidobacteriaceae compared to their healthy peers [46]. Additionally, families such as Erysipelotrichaceae, Ruminococcaceae, and Sutterellaceae are more prevalent in children with food allergies than in those with atopic dermatitis, and their abundance correlates with IgE levels [46].

Children with IgE-mediated food hypersensitivity (FH) also exhibit decreased gut microbial diversity and richness, accompanied by an increased abundance of Firmicutes and a relative underrepresentation of Bacteroidetes [47]. Moreover, food-allergic children display elevated levels of pro-inflammatory or potentially pathogenic genera such as Parabacteroides, Blautia, Escherichia-Shigella, Ruminococcus gnavus (R gnavus). At the same time, beneficial taxa including Bifidobacterium, Clostridium, Bacteroides (e.g., *B. dorei*, *B. vulgatus*), and Bifidobacterium longum are significantly reduced [36,39,43].

Infants with non-IgE-mediated cow’s milk protein allergy (CMPA) exhibit a higher abundance of Eggerthellaceae, Rothia, Lachnospiraceae, and Peptostreptococcaceae, and a lower abundance of Bifidobacteria. Eggerthellaceae was also more abundant in the breast milk samples of mothers whose infants had CMPA [30].

Notably, strains of R. gnavus identified in allergic children exhibit a diminished capacity for fiber degradation and harbor genes encoding a pro-inflammatory polysaccharide [36].

Specific increases in Ruminococcaceae, Clostridiaceae, and Erysipelotrichaceae have been observed in children with milk hypersensitivity; however, Clostridiaceae and Erysipelotrichaceae are also elevated in peanut hypersensitivity [47]. Egg white hypersensitivity was associated with increases in Clostridiaceae, Lachnospiraceae, and Pasteurellaceae [47]. Children with peanut allergy also exhibit reduced gut microbial diversity and richness, characterized by increased Bacteroides and Klebsiella, and lower levels of beneficial taxa, such as Faecalibacterium, Bifidobacterium, and Lachnospiraceae [50]. These children also exhibit elevated levels of inflammatory cytokines (IL-4, IL-5, and IL-13) and reduced numbers of regulatory T cells (Treg cells), with a strong positive correlation between Bacteroides abundance and levels of IL-4 and IL-5 [50].

Japanese infants who developed allergies by 24 months exhibited a significant reduction in early gut microbiota community structure, particularly in beta diversity, and a decrease in Bifidobacterium occupancy during the pre-weaning period (1–6 months) compared to their non-allergic peers. The study identified six distinct neonatal gut microbiome enterotypes, with Bifidobacterium-dominant enterotypes characterized by higher fecal propionate concentrations, associated with the lowest risks of developing food sensitization (FS) and FA, especially hen egg white sensitization, at 2 years old in one cohort and 9 months old in another [49].

Children with CMPA on a cow’s milk protein-free (CMPF) diet showed a decrease in Bifidobacterium levels and an increase in Clostridioides and Escherichia-Shigella. These microbial shifts persist even after tolerance is acquired and are not reversed by probiotic administration [44]. Early childhood increases in Escherichia-Shigella and reductions in Bifidobacteria have been linked to persistent allergic symptoms beyond two years of age [44].

Children who later developed allergic conditions showed a lower proportion of IgA-bound fecal bacteria at 12 months of age compared to healthy controls, independent of total IgA concentrations or overall bacterial load [26]. Moreover, the specific bacterial targets of early IgA responses, as well as the overall pattern of IgA recognition, differed between healthy and allergic children and could be detected as early as one month of age [26].

Allergic children also exhibited a heightened pro-inflammatory potential, with increased gene abundance for bacterial lipopolysaccharide (LPS) and urease, and decreased fecal levels of short-chain fatty acids (SCFAs [36]). Fecal supernatants from children elicited enhanced T2 cytokine responses (IL-5, IL-13) from CD4+ T cells. Furthermore, the gut microbiome of children with both food allergies and atopic dermatitis exhibits a greater inflammatory potential compared to that of healthy children [36].

The gut microbiome of children with food allergies and malnutrition (FAM) showed an increase in specific genera, such as Alistipes and Parabacteroides, compared to healthy controls. In contrast, FA children without malnutrition (FANM) showed an increase in alpha diversity [51]. There was a positive correlation between the relative abundance of Faecalibacterium and total IgE levels. Researchers identified 14 pivotal microbial markers with substantial classification potential for differentiating between FA children with and without malnutrition and healthy controls, suggesting that gut microbiota analysis could be a noninvasive method for identifying potential markers and indicating the need for tailored microbiota-targeted therapies.

### 4.6. Dietary Modulation of Gut Microbiota in Allergic Individuals: The Role of Prebiotics and Probiotics

The growing body of evidence implicating intestinal dysbiosis as a key etiological factor in the development of allergic disorders has led to considerable interest in the therapeutic potential of dietary interventions, including the use of prebiotics and probiotics, for managing and potentially preventing such conditions.

#### 4.6.1. Diet and Prebiotics

Diet plays an important role in shaping the composition of the gut microbiota [100]. Specific dietary constituents—particularly dietary fiber, polyphenols, and fermented foods—are known to support the proliferation of health-promoting bacterial taxa [101,102]. Notably, these substrates stimulate the growth of SCFA-producing genera, such as Bifidobacterium and Faecalibacterium, which contribute to gut barrier integrity, mucosal immune regulation, and the expansion of regulatory T cells (Tregs) [103].

In contrast, Western dietary patterns—typically characterized by high intake of saturated fats and refined sugars, alongside low fiber consumption—have been linked to dysbiotic microbial communities with pro-inflammatory profiles, potentially exacerbating allergic conditions [101].

Early-life dietary practices, including breastfeeding and the timely introduction of allergenic foods during critical windows of immunological development, have shown promise in modulating the gut microbiota and reducing the risk of allergies [11,12].

Prebiotics, primarily short-chain oligosaccharides and polysaccharides, serve as fermentable substrates for beneficial gut bacteria. Among the most extensively studied are inulin and fructooligosaccharides (FOS), belonging to the fructan family, and galactooligosaccharides (GOS), derived from galactans [104]. Human milk itself provides a rich source of prebiotics, particularly human milk oligosaccharides (HMOs). Interventional studies supplementing inulin, FOS, or GOS have consistently reported increased abundance of Bifidobacteria, Lactobacilli, Akkermansia, and Roseburia—taxa associated with improved gut health and immune function [105,106,107].

Moreover, maternal dietary intake during pregnancy has been correlated with the presence of specific microbial taxa in both maternal and neonatal microbiota, including Collinsella, Lachnospira, Sutterella, and Faecalibacterium, as well as the Firmicutes/Bacteroidetes ratio [108]. However, not all studies have demonstrated robust microbiota shifts; for instance, Dawson et al. reported minimal compositional changes in maternal or infant microbiota following increased prebiotic and probiotic intake, suggesting that baseline health status may modulate responsiveness to dietary interventions [33].

In non-breastfed infants with suspected CMPA, supplementation with extensively hydrolyzed casein formula (EHCF) enriched with HMOs such as 2′-fucosyllactose (2′FL) and lacto-N-neotetraose (LNnT) resulted in favorable shifts in microbial diversity and a rise in Bifidobacteria (notably B. bifidum and B. breve), accompanied by decreased abundance of potentially pathogenic taxa, including Enterobacteriaceae and Clostridium sensu stricto [37]. Similarly, an amino acid-based formula (AAF) supplemented with a synbiotic mixture significantly improved microbiota composition in allergic infants, restoring Bifidobacteria levels to those typically observed in breastfed infants [28].

#### 4.6.2. Probiotics

Probiotics—defined as live microorganisms that confer health benefits to the host when administered in adequate amounts—represent a promising adjunctive strategy in allergy management. Among the most extensively investigated genera are Lactobacillus and Bifidobacterium, which encompass strains with potent immunomodulatory and gut-barrier-reinforcing properties [109]. These organisms can attenuate the T2-skewed immune phenotype frequently observed in allergic individuals by promoting a more balanced T1/T2 response and by enhancing epithelial barrier function and dendritic cell tolerogenicity [110].

Numerous clinical and preclinical studies have been conducted to investigate the role of probiotics in preventing and managing food allergies.

One notable example is early-life supplementation with Escherichia coli O83:H31 (EcO83) in neonates born to mothers with allergies, which resulted in a reduced incidence of allergic manifestations by age 10 despite minimal long-term changes in gut microbiota composition. Mechanistically, EcO83 modulates dendritic cell behavior by upregulating CD83 and enhancing IL-10 secretion, thereby supporting a tolerogenic immune environment [41]. Similarly, the use of EHCF supplemented with Lactobacillus rhamnosus GG (LGG) in children with IgE-mediated CMA was associated with a reduced incidence of additional allergic conditions and accelerated acquisition of oral tolerance over 36 months [27,53]. Longitudinal follow-up suggests that this intervention not only improves immunological tolerance but also reduces the risk of functional gastrointestinal disorders in later childhood [53].

Supplementation with Bifidobacterium bifidum TMC3115 in non-breastfed infants with CMA demonstrated reductions in allergic symptom scores across multiple domains—gastrointestinal, respiratory, dermatological, and systemic—alongside immune modulation marked by increased IL-10, decreased pro-inflammatory cytokines (TNF-α, IL-1β, IL-6), lowered IgE, and elevated IgG2 levels [31]. These effects were mirrored by increases in microbial alpha diversity and the relative abundance of beneficial taxa, including Turicibacter, Sutterella, and Parabacteroides.

Furthermore, combining Lactiplantibacillus plantarum with oral immunotherapy (OIT) resulted in superior outcomes in children with CMA, characterized by enhanced rates of tolerance acquisition and favorable immunologic shifts, including increased β-lactoglobulin-specific IgG4 and reductions in IL-5 and IL-9 [38]. The presence of a Bifidobacterium-dominant microbiota module (Mb-24) has also been identified as a potential predictive biomarker for successful OIT outcomes, with sustained unresponsiveness being more likely in individuals with this microbial signature [42].

EHCF supplemented with Lactobacillus rhamnosus GG (LGG) further led to increased fecal butyrate and enrichment of butyrate-producing genera, such as Blautia and Roseburia, supporting the hypothesis that microbial metabolic outputs mediate many of the observed clinical benefits [27].

Maternal supplementation with prebiotics during pregnancy has also emerged as a promising window of opportunity to influence the offspring’s gut microbiota and immune trajectory favorably. One study reported increased Bifidobacteria in the maternal gut and decreased Negativicutes in both the mother’s and infant’s microbiota following such intervention [48]. However, more robust evidence is needed to define the optimal timing, type, and dosage of supplementation.

Together, these data highlight the intricate interplay between diet, gut microbiota, and immune function in the context of allergic disease. While current evidence supports the utility of prebiotic and probiotic interventions, particularly during early developmental windows, ongoing high-quality clinical trials are needed to refine strain-specific efficacy, identify responders, and establish long-term benefits.

## 5. Conclusions

Evidence synthesized in this review emphasizes the central role of gut microbiota in shaping immune tolerance to dietary antigens.

Mode of delivery, infant feeding practices, antibiotic exposure, and environmental microbial diversity all play a significant role in early microbial colonization. Perturbations in microbial composition, especially the depletion of beneficial taxa such as *Bifidobacteria*, *Clostridia*, and *Faecalibacteria*, are consistently associated with an increased susceptibility to allergic sensitization and clinical food allergy.

The gut microbiota exerts immunomodulatory effects through multiple pathways, including the promotion of regulatory T cell differentiation, the production of anti-inflammatory cytokines like interleukin-10, and the synthesis of short-chain fatty acids that influence epithelial integrity and immune homeostasis. Distinct microbial and metabolic signatures observed in individuals with allergies suggest potential diagnostic and prognostic applications, as well as opportunities for targeted therapeutic interventions.

Dietary modulation of the gut microbiota through prebiotic and postbiotic supplementation demonstrates potential efficacy in promoting immune tolerance. However, studies show substantial heterogeneity regarding methodology, intervention protocols, targeted population, and microbial strains. Therefore, further studies are necessary to determine strain-specific efficacy, optimal windows of intervention, and long-term immunological outcomes.

## Figures and Tables

**Table 1:** Quality assessment of included studies.

Author (Year)	Article Type	Quality of Article	Justification-Limitations
Canani et al. (2016) [25]	Controlled intervention study	Fair	Controlled intervention (lack of clear randomization and blinding details), coupled with a very small sample size for the intervention groups.
Dzidic et al. (2017) [26]	Prospective observational cohort study	Fair	No explicit reporting of participation rate for this specific sub-study, lack of details for blinding procedures for outcome assessment, no information on loss to follow-up for the selected cohort, small sample size.
Canani et al. (2017) [27]	Randomized Controlled Trial	Good	Strong methodology. Limitations: parents of the participants were aware of the assigned treatment, which could potentially introduce reporting bias. The study population was limited to children with immunoglobulin E (IgE) -mediated cow’s milk allergy from specific socioeconomic and urban backgrounds, which may restrict the generalizability of the results.
Candy et al. (2018) [28]	Randomized control trial	Good	Good methodology. Limitations: Absence of a standardized diagnostic test or mandatory food challenge for non-IgE cow’s milk allergy, potential baseline imbalances in delivery mode, and the exploratory nature of its clinical outcomes, meaning the study was not powered to show significant clinical benefits.
Savage et al. (2018) [11]	Observational cross-sectional study	Fair	The observational design precludes establishing causality. The self-reported nature of dietary data, particularly the retrospective assessment of infant diet at the time of stool collection, introduces a risk of misclassification and recall bias. Specific recruitment criteria (family history of allergy/asthma) may limit the generalizability of the findings to broader populations.
Nocerino et al. (2019) [29]	Prospective cohort study	Good	Strong methodological quality, well designed and executed. Observational study—cannot establish causality (a fundamental characteristic of its design, not a flaw in execution). The large sample size and clear presentation of results further support its classification as a high-quality study.
Aparicio et al. (2020) [30]	Observational pilot cohort study	Fair	Small sample size of 30 mother–infant pairs, which limits the statistical power. The objectives are clearly stated and the methodology for sample collection, molecular, and immunological analyses is well-described, ensuring internal validity for the conducted tests.
Jing et al. (2020) [31]	Randomized double-blind control trial	Good	Good methodology. Conducted at a single center, potentially limiting generalizability.
Bao et al. (2021) [32]	Cross-sectional study	Good/ Fair	Lacks explicit details on sample size justification and blinding of outcome assessors. Cannot establish temporality of exposure and outcome due to cross-sectional design.
Dawson et al. (2021) [33]	Randomized control trial	Fair	Small sample size. The reliance on self-reported dietary data, lack of full blinding, and per-protocol analysis.
Marrs et al. (2021) [12]	Randomized control trial	Good	Good methodology. No sample size justification for microbiome analysis. Randomization concealment methods not described. Not all enrolled participants followed for full duration of microbiome study. Not explicitly stated if microbiome/clinical outcome assessors were blinded. Did not discuss/quantify potential inter-group contamination. Direct clinical importance of microbiota changes in preventing food allergy not established.
Nocerino et al. (2021) [34]	Prospective cohort study	Good	Good methodology. Participants not randomized to formula groups, continued prescribed formula. Residual confounding cannot be entirely ruled out.
Homann et al. (2021) [35]	Longitudinal cohort study	Good	Good methodology. The comparison of two geographically distinct cohorts adds to the robustness of the findings, and the authors acknowledge potential limitations (the relatively small sample size and differences in dietary introduction approaches between cohort)
De Filippis et al. (2021) [36]	Randomized double-blind control trial	Good	Good methodology. Absence of reported participation rate, sample size justification, and explicit statement on outcome assessor blinding.
Boulange et al. (2023) [37]	Single-arm, prospective clinical study	Fair	Lack of control group (changes could be due to natural infant gut maturation/environmental factors). No sample size justification, small subject number. Approximately 21% participant dropouts. Limited clinical importance (did not directly assess clinical outcomes). Potential for bias (no explicit blinding for lab analysis, manufacturer funding).
Hanada et al. (2023) [38]	Randomized controlled trial	Fair	The study explicitly states it is a pilot study and was not powered for its primary clinical outcome, which was indeed found to be non-significant. The lack of explicit detail on controlling for co-interventions is also a minor concern.
Yan et al. (2023) [39]	Longitudinal observational study	Fair	Small sample size, fecal samples were not collected at the 2-year follow-up, which prevented establishing direct connections between microbiota shifts and symptom resolution or persistence post-follow-up.
Gao et al. (2023) [40]	Prospective cohort study	Good	Rigorous methodology, combined with a substantial sample size across two distinct populations, enhances the clinical relevance of the conclusions.
Sukenikova et al. (2023) [41]	Prospective cohort study	Good	Good methodology, substantial 10-year follow-up. The use of both clinical allergy diagnoses (allergist confirmation) and parental reports for allergy status, combined with in vitro immunological assays and gut microbiota analysis (16S rRNA gene sequencing), demonstrates a multi-faceted approach to evaluating the intervention.
Shibata et al. (2024) [42]	Ancillary cohort study	Fair	Small sample size, no multivariable models, no adjustment for confounding factors. Lack of detail regarding participation rate.
Hara et al. (2024) [43]	Case–Control	Fair	Clear objectives and a well-defined case–control design appropriate for investigating associations. However, as a case–control study, it cannot establish causality. The sample size of 130 participants is moderate for gut microbiome studies. The study’s focus on a single age group and recruitment from one institution might limit the generalizability of its findings.
Castro et al. (2024) [44]	Prospective longitudinal cohort study	Fair	Small sample size, which restricts the generalizability and statistical power of the findings. The absence of a control group of healthy children makes it challenging to draw robust conclusions about the observed gut microbiota changes.
Korpela et al. (2024) [45]	Prospective observational study	Good	The sample size is substantial, the methodology is robust. The comparison with pre-pandemic cohorts strengthens the study’s ability to assess the impact of social distancing
Nekrasova et al. (2024) [46]	Observational case- control	Good	The study is well-designed as a case–control study. A comprehensive set of statistical analyses were utilized to process the complex metagenomic data. The researchers applied data transformation & quality control measures to mitigate sampling and rarefaction bias.
Chen et al. (2024) [47]	Observational cross-sectional	Good	Good methodology. The study focused on children aged 18 to 36 months, which might limit the generalizability of the findings to older children or adults.
Jones et al. (2024) [48]	Randomized placebo-controlled trial	Good	The study employed a strong randomized, double-blinded, placebo-controlled design, which minimizes bias. The interventions and outcome measures were clearly defined, and robust methodologies were used for microbiome sequencing and SCFA quantification. Statistical analyses were comprehensive and included appropriate corrections for multiple comparisons, and the laboratory analyses were performed while blinded to treatment allocation.
Shibata et al. (2025) [49]	Combined analysis of two longitudinal birth-cohort studies	Good	Robust design by combining data from two prospective longitudinal birth-cohort studies, allowing for comprehensive analysis of gut microbiota over time. Limitations: while the study combined two cohorts, certain relationships between gut microbiota and outcomes showed heterogeneity and were not consistently shared between the two studies, except for *Bifidobacterium*.
Li et al. (2025) [50]	Case- Control	Fair	Cross-sectional nature, preventing the establishment of causal relationships. The relatively small sample size and recruitment from a single center might limit the generalizability of the results. Furthermore, the study did not delve into the functional aspects of the gut microbiota and did not fully control for confounding factors like diet, which could significantly influence gut microbial composition.
Zhang et al. (2025) [51]	Cross-Sectional study	Good	Clear research question, an appropriate cross-sectional design for its objectives, well-defined participant groups with matching, and detailed, ethically approved methods, robust statistical analyses. Limitations: cross-sectional design, which prevents establishing causal relationships. It also did not account for confounding factors like daily diet, activity levels, or lifestyle.
Imoto et al. (2025) [52]	Prospective cohort study	Good	Robust prospective cohort design, clear objectives, and detailed methodology for sample collection and statistical analyses demonstrate scientific rigor. The study’s primary limitation is the focus on specific genes (16S rRNA), which only reveals composition rather than functional capabilities of the microbiota.
Nocerino et al. (2025) [53]	Prospective cohort study	Good	Strong prospective cohort design with a lengthy 6-year follow-up, robust methodology. Limitations: no formal sample size calculation was performed specifically for this 72-month follow-up, as it extended a previous 36-month study

Legend: 16S rRNA—16S ribosomal RNA; SCFA—short-chain fatty acid.

**Table 2:** Observational studies included in the review.

Author (Year)	Population Studied	Number of Subjects	Key Findings
Dzidic et al. (2017) [26]	Children from the LISA birth cohort in Sweden followed prospectively for the first 7 years of life	28 children	Children who developed allergic conditions showed a lower proportion of IgA-bound fecal bacteria at 12 months of age, independent of total IgA levels or overall bacterial load. Specific bacterial targets of early IgA responses and the overall IgA recognition patterns varied between healthy and allergic children, with these altered patterns detectable as early as 1 month of age.
Savage et al. (2018) [11]	Infants participating in a cohort selected based on parental history of asthma or allergy.	323 infants included in the primary analyses: exclusively breastfed: 95 infants, exclusively formula-fed: 169 infants, mixed feeding: 43 infants	Breastfeeding is the most significant dietary factor influencing the infant intestinal microbiome, leading to higher levels of beneficial bacteria like *Bifidobacterium*, *Lactobacillus*, and *Clostridia*. Breastfed infants had lower microbial diversity, and the impact of breastfeeding on the microbiome varied by the child’s race/ethnicity.
Aparicio et al. (2020) [30]	Mother–infant pairs where the infants were diagnosed with colic, (CMPA), or proctocolitis, healthy control infants.	30 mother–infant pairs divided into four groups: Colic, Non-IgE-mediated CMA, Healthy Controls	Infants with non-IgE-mediated CMPA showed a higher abundance of *Eggerthellaceae*, *Rothia*. *Lachnospiraceae*, *Peptostreptococcaceae*, and a lower abundance of *Bifidobacterium* *Eggerthellaceae* more abundant in the breast milk samples of mothers whose infants had CMA
Bao et al. (2021) [32]	Twin pairs (discordant/concordant for food allergy)	18 twin pairs	Identified a bacterial signature distinguishing food-allergic from healthy individuals Reduced fecal microbiota richness in food-allergic individuals. Higher *Phascolarctobacterium faecium* and *Ruminococcus bromii* in healthy twins. Lower diacylglycerol in food-allergic twins Differences in microbiome/metabolome correlated with food allergy clinical severity.
Marrs et al. (2021) [12]	Exclusively breastfed infants aged between 12 and 17 weeks at enrollment.	Provided baseline (3-month) stool samples for microbiome analysis: 288 infants Subset for longitudinal microbiome analysis (samples at 3, 6, and 12 months): 70 individuals.	At 3 months, exclusively breast-fed infants’ gut microbiota was heterogeneous (*Bifidobacterium*-rich, *Bacteroides*-rich, *Escherichia/Shigella*-rich clusters). Over first year, infant gut microbial communities mature towards Bacteroides-rich, adult-like patterns. Mode of delivery significantly influenced 3-month microbiome (fewer *Bacteroides* in C-section). C-section associated with reduced gut microbiota diversity at 3 months. Increased *Clostridium sensu stricto* at 3 months associated with atopic dermatitis at 3 and 12 months. Other genera (*Haemophilus*, *Veillonella*) linked to atopic dermatitis. Early introduction of allergenic solids from 3 months significantly increased Shannon microbial diversity at 6 months. No evidence that specific microbiota changes due to early allergenic food introduction were associated with food allergy/sensitization development.
Homann et al. (2021) [35]	Healthy, full-term, vaginally born infants	24 infants	Microbial richness and diversity in the infant gut increased over time and were positively associated with dietary diversity. *Bifidobacterial* taxa were positively associated with dietary diversity, while *Veillonella* taxa showed a negative association.
De Filippis et al. (2021) [36]	Children with diagnosed IgE-mediated food allergies (FA) or respiratory allergies (RA) and healthy controls (CT)	Initially evaluated: 90 subjects with allergy and 30 healthy controls (Total = 120). Included in shotgun metagenomics analysis 114 subjects	Allergic children have higher *Ruminococcus gnavus* (*R. gnavus*) and *Faecalibacterium prausnitzii*, and depletion of beneficial bacteria (*Bifidobacterium longum*, *Bacteroides dorei*, *B. vulgatus*, fiber-degrading taxa). Strains of *R. gnavus* in allergic children showed reduced fiber degradation and genes for a pro-inflammatory polysaccharide. Allergic children showed increased pro-inflammatory potential (enriched genes for bacterial lipopolysaccharides and urease) and had lower fecal levels of SCFAs. Fecal supernatants from allergic children (FA and RA) elicited increased pro-allergic Type 2 (T2) cytokines (IL-5, IL-13) from CD4+ T cells.
Yan et al. (2023) [39]	Children with FA and controls	10 children aged 0 to 3 years with FA and 10 controls	FA children showed increased levels of *Akkermansia*, *Parabacteroides*, *Blautia*, and *Escherichia-Shigella*, while *Bifidobacterium* and *Clostridium* were decreased. Early childhood increases in *Escherichia-Shigella & reductions* in *Bifidobacterium* were associated with persistent allergic symptoms after 2 years.
Gao et al. (2023) [40]	Infants from the Barwon Infant Study (BIS) cohort and The Copenhagen Prospective Studies on Asthma in Childhood (COPSAC2010) cohort from Denmark	208 infants from BIS cohort, 200 infants from COPSAC2010 cohort	Having siblings in early life significantly reduces the risk of developing food allergy by accelerating gut microbiota maturity (higher microbial alpha diversity and increased concentrations of SCFAs)
Shibata et al. (2024) [42]	Children 1 week- 7 years old & their mothers from two distinct birth cohorts. The CHIBA study focused on a high-risk cohort with a family history of allergic diseases, while the Katsushika study was originally a randomized trial evaluating skincare +/− synbiotics	270 participants in the CHIBA study & 245 participants in the Katsushika study A total of 2563 fecal microbiome samples were analyzed from these children and their mothers	Six distinct neonatal gut microbiome enterotypes revealed through clustering, revealing age-dependent maturation patterns More gut bacteria, particularly *Bifidobacterium*, in 1-month-old children showed significant relationships with the development of food sensitization (FS) and FA than in 1-week-old children *Bifidobacterium*-dominant enterotypes, characterized by higher fecal propionate concentrations, were associated with the lowest risks of developing FS and FA, especially hen egg white sensitization, at 2 years old in one cohort and 9 months old in another
Hara et al. (2024) [43]	One-and-a-half-year-old food-allergic and healthy children.	130 participants: 65 FA children & 65 healthy control children	FA children had a significantly lower abundance of *Bacteroides* and a higher abundance of *Clostridium sensu stricto 1* and *Enterococcus*. While no significant differences in alpha diversity were observed, beta diversity analysis showed a clear distinction between the two groups.
Castro et al. (2024) [44]	Pediatric patients with IgE-mediated or non-IgE-mediated CMA, aged 0–12 months a	26 pediatric patients diagnosed with CMPA who followed a cow’s milk protein-free (CMPF) diet	Children with CMA on a CMPF diet showed a decrease in *Bifidobacterium* and an increase in *Clostridioides* and *Escherichia-Shigella*. These changes were not reversed by the introduction of probiotics. Diet-induced alterations persisted, even after the acquisition of tolerance to cow’s milk.
Korpela et al. (2024) [45]	Irish infants born between March and May 2020, during the initial phases of the COVID-19 pandemic & associated social distancing restrictions	360 infants	Infants born during pandemic-related social distancing had higher *Bifidobacteria* and lower environmental bacteria like *Clostridia* compared to pre-pandemic cohorts Prevalence of allergy and atopic dermatitis did not increase Diet, particularly breastfeeding and plant-based weaning foods, significantly shaped the microbiota Higher *Bifidobacteria* levels at 6 months and a greater relative abundance of butyrate producers at 12 months were negatively associated with atopic dermatitis
Nekrasova et al. (2024) [46]	Children with atopic dermatitis (AD) and food allergies (FA)	128 children aged 3 to 12 years, divided into three groups: AD Group: 49 children, FA Group: 47 children, Control Group: 32 healthy volunteers.	Both AD & FA groups showed an abundance of *Pasteurellaceae* and *Erysipelotrichaceae* families & a decrease in *Barnesiellaceae* compared to healthy participants Children with FA had a decrease in *Desulfovibrionaceae* and an enrichment of *Bifidobacteriaceae* compared to healthy participants Comparing FA patients to AD patients, *Erysipelotrichaceae*, *Ruminococcaceae*, and *Sutterellaceae* were more prevalent in the FA group The representation of *Ruminococcaceae* and *Sutterellaceae* families was associated with IgE levels
Chen et al. (2024) [47]	Children with different IgE-mediated food hypersensitivity (FH)	Fecal samples from: 57 children with IgE-mediated hypersensitivity (FH) & 24 healthy children aged 18 to 36 months.	Children with IgE-mediated FH (milk, egg white, soy) had lower gut microbiota diversity and richness compared to healthy children. They also showed higher abundances of *Firmicutes* and an underrepresentation of *Bacteroidetes*. Specific increases in relative abundances of *Ruminococcaceae*, *Clostridiaceae*, and *Erysipelotrichaceae* were observed in children with milk hypersensitivity, while *Clostridiaceae* and *Erysipelotrichaceae* were increased in peanut hypersensitivity. Egg white hypersensitivity was associated with increases in *Clostridiaceae*, *Lachnospiraceae*, and *Pasteurellaceae*.
Zhang et al. (2025) [51]	80 children with FAs, 40 healthy controls	40 children with FAs and adequate weight (FANM) 40 children with food allergies and malnutrition (FAM) 40 healthy control children (matched for age and sex)	FA children without malnutrition (FANM) showed an increase in alpha diversity, while the FA children with malnutrition (FAM) exhibited an increase in specific genera like *Alistipes* and *Parabacteroides*. Positive correlation between the relative abundance of the genus *Faecalibacterium* and total IgE levels. Identified 14 pivotal microbial markers to differentiate between FA children with and without malnutrition from healthy controls.
Imoto et al. (2025) [52]	Cohort of Japanese infants from birth through 24 months of age	121 infants	Antimicrobial exposure at delivery and the presence of older siblings significantly altered the gut microbiota composition and overall diversity in early infancy. Infants who developed allergies by 24 months exhibited significant shifts in early gut microbiota community structure, particularly in beta diversity, and reduced *Bifidobacterium* occupancy during the pre-weaning period (1–6 months) compared to non-allergic peers.
Li et al. (2025) [50]	Pediatric patients with peanut allergy and healthy controls	97 children were included in the study, comprising: 35 children with peanut allergy (PA group) & 62 healthy children (HC group)	Reduced gut microbial diversity and richness in PA group compared to HC. *Bacteroides* and *Klebsiella* were significantly enriched in the PA group while *Faecalibacterium*, *Bifidobacterium*, and *Lachnospiraceae* were less abundant. PA group showed higher levels of inflammatory cytokines (IL-4, IL-5, IL-13) and lower levels of regulatory T cells (Treg cells). Strong positive correlation between *Bacteroides* abundance & IL-4 and IL-5 levels

IgA: immunoglobulin A, IgE: immunoglobulin E, IL: interleukin, CMA: cow’s milk allergy, CMPA: cow’s milk protein allergy, SCFA: short-chain fatty acid, FA: food allergy, RA: respiratory allergy, FS: food sensitization, IL: interleukin, CMPF: cow’s milk protein-free, AD: atopic dermatitis, FANM: food allergic children without malnutrition, FAM: food allergic children with malnutrition, PA: peanut allergy, FH: food hypersensitivity, HC: healthy children, Treg: regulatory T cells.

**Table 3:** Experimental studies included in the review.

Author (Year)	Population Studied	Number of Subjects	Key Findings
Canani et al. (2016) [25]	Infants with CMA	19 infants with CMA in test group 20 healthy controls	CMA infants: gut microbiota dominated by *Lachnospiraceae* and *Ruminococcaceae* EHCF + LGG increased abundance of *Blautia, Roseburia, Coprococcus* Tolerance linked to butyrate increase and enrichment of *Blautia* and *Roseburia* (strain-level differences)
Canani et al. (2017) [27]	Children with IgE-mediated CMA, aged 1–12 months	EHCF group: 110 children EHCF supplemented with *L. rhamnosus* GG (EHCF + LGG): 110 children	EHCF + LGG significantly reduced the occurrence of other allergic manifestations (such as eczema, urticaria, asthma, and rhinoconjunctivitis) over 36 months in children with IgE-mediated CMA EHCF + LGG hastened development of oral tolerance to cow’s milk
Candy et al. (2018) [28]	Infants with suspected non-IgE-mediated CMA	35 infants in test group receiving AAF with synbiotic 36 controls receiving AAF 51 breastfed infants	The synbiotic-containing AAF significantly increased fecal *Bifidobacteria* levels The beneficial changes in *Bifidobacteria* levels observed in the test group were comparable to those found in healthy breastfed infants
Nocerino et al. (2019) [29]	Children aged 4–6 years with IgE-mediated CMA in their first year of life who had acquired oral tolerance to cow’s milk proteins for at least 12 months, healthy controls	EHCF cohort: 110 children EHCF + LGG cohort: 110 children Healthy Cohort: 110 healthy children	Children treated with EHCF + LGG in infancy had lower incidence of functional gastrointestinal disorders (FGIDs) later in childhood compared to those treated with EHCF alone. The prevalence of FGIDs in the EHCF + LGG group was similar to that in healthy controls.
Jing et al. (2020) [31]	Infants with CMA who could not be exclusively breastfed	128 in test group, 128 in control group	*B. Bifidum TMC3115* intervention: - reduced allergic scores across GI, respiratory, skin, and whole-body reactions - decreased pro-inflammatory cytokines (TNFα, IL-1β, IL-6), increased anti-inflammatory IL-10. - reduced serum IgE, increased serum IgG2 - increased abundance/diversity of beneficial bacteria (*Bifidobacterium*, *Lactobacillus*, *Turicibacter*, *Sutterella*, *Parabacteroides*). Reduced proportion of certain *Firmicutes* genera. - increased alpha-diversity scores of gut microbiota Serum IgE/IgG2 levels strongly correlated with gut microbiota alpha-diversity
Dawson et al. (2020) [33]	Healthy pregnant women from gestation week 26	45 women randomized: 22 in control group 23 in intervention group	Dietary intervention led to a significant increase in dietary variety and consumption of prebiotic and probiotic foods in the intervention group compared to the control group. No significant changes obtained in the composition (diversity or specific bacterial taxa) of either maternal or infant gut microbiota at 6 weeks postpartum.
Nocerino et al. (2021) [34]	Non-breastfed infants (aged 1–12 months) with confirmed IgE-mediated CMA	Enrolled into the study: 365 subjects: 73 subjects in each of the 5 formula cohorts (EHCF + LGG, rice hydrolyzed formula, soy formula, EHWF, or AAF)	Other atopic manifestations (eczema, urticaria, asthma, rhinoconjunctivitis) significantly lower in EHCF + LGG cohort. 36-month immune tolerance acquisition rate significantly higher in EHCF + LGG cohort. EHCF + LGG cohort showed greater increase in immune tolerance to cow’s milk proteins over 36 months.
Boulange et al. (2023) [37]	Non-breastfed infants aged between 2 weeks and 6 months with symptoms suggestive of CMA	194 non-breastfed infants: 97 to test formula 97 to control formula	Supplemented EHCF (with 2′FL and LNnT) increased alpha diversity and shifted beta diversity towards healthy, breastfed infant profiles after 3 months Significant increase in *Bifidobacterium* (especially *B. bifidum*, *B. breve*) and decrease in potentially pathogenic bacteria (*Enterobacteriaceae, Clostridium sensu stricto*) Fecal metabolome changed significantly (increased SCFAs, alterations in amino acid, bile acid, carbohydrate metabolism)
Hanada et al. (2023) [38]	Children aged 1–18 years with IgE-mediated CMA diagnosed by oral-milk challenge test	31 children assigned to the Intervention group (probiotic *Lactiplantibacillus plantarum*—LP and oral immunotherapy-OIT) 30 to the control group (receiving only OIT)	The LP group showed a higher rate of improved cow milk tolerance (41.4%) compared to the control group (37.9%) after 24 weeks. The LP group exhibited favorable changes in serum biomarkers (higher β-lactoglobulin-IgG4 and lower IL-5 and IL-9) compared to the control group.
Sukenikova et al. (2023) [41]	Pregnant women and their neonates, divided into groups based on maternal allergic status and probiotic supplementation.	Probiotic-supplemented children of allergic mothers: 56, Non-supplemented children of allergic mothers: 57, Non-supplemented children of healthy mothers: 45	Early postnatal supplementation with *Escherichia coli* 083:K24:H31 (EcO83) in newborns of allergic mothers was associated with a decreased incidence of allergy at 10 years of age, comparable to that of low-risk children (children of healthy mothers). While the probiotic did not significantly alter the gut microbiota composition at 10 years of age, in vitro experiments showed that EcO83 stimulation increased CD83 expression and interleukin (IL)-10 secretion by cord blood dendritic cells.
Shibata et al. (2024) [49]	School-age children with IgE-mediated CMA undergoing oral OIT	32 children Completed OIT and assessed for sustained unresponsiveness (SU): 28 children	7 of 28 participants (22%) completing OIT achieved sustained unresponsiveness. Lower milk- and casein-specific IgE at baseline and throughout OIT associated with higher probability of SU. Higher *Bifidobacterium*-dominant module (Mb-24) at baseline and longitudinally associated with greater chance of achieving SU. Gut microbiota and fecal water-soluble metabolite (WSM) profiles showed temporary changes at OIT start but returned to baseline by end. Alpha diversity decreased initially but recovered. Milk- and casein-specific IgE levels negatively correlated with several *Lachnospiraceae* and *Streptococcaceae* gut microbiota modules.
Jones et al. (2024) [48]	Pregnant women (recruited before 21 weeks’ gestation) and their infants up to 12 months of age	74 mother–infant pairs	Maternal prebiotic supplementation during pregnancy and lactation significantly modified both the maternal and developing infant gut microbiome. Infant microbial beta-diversity significantly differed between prebiotic and placebo groups at 12 months of age. Increased abundance of *Bifidobacteria* in the maternal microbiota and a reduction in *Negativicutes* in both maternal and infant microbiota after supplementation. Significant changes in short-chain fatty acid (SCFA) concentrations were observed, including an increase in acetic acid in the prebiotic group from 20 to 28 weeks’ gestation.
Nocerino et al. (2025) [53]	Non-breastfed infants aged 1–12 months with immunoglobulin E (IgE)-mediated CMA	313 subjects divided in five groups: EHCF + LGG (*n* = 64), Rice hydrolyzed formula RHF (*n* = 62), Soy formula SF (*n* = 63), extensively hydrolyzed whey formula EHWF (*n* = 60), AAF (*n* = 64)	EHCF + LGG diet was associated with a significantly lower incidence of other atopic manifestations and a higher rate of immune tolerance acquisition in children with CMA. The 72-month immune tolerance acquisition rate was also higher in the EHCF + LGG group.

IgE: immunoglobulin E, IgG: immunoglobulin G, CMA: cow’s milk allergy, EHCF + LGG: extensively hydrolyzed cow’s milk formula with L. rhamnosus GG, EHWF: extensively hydrolyzed whey formula, SCFA: short-chain fatty acid, AAF: amino-acid based formula, FGID: functional gastro-intestinal disorders, LP: Lactiplantibacillus plantarum, OIT: oral immunotherapy.
